# Transcatheter Caval Implantation for Severe Tricuspid Regurgitation

**DOI:** 10.1007/s11886-024-02190-8

**Published:** 2025-01-07

**Authors:** Vincent Chen, Omar Abdul-Jawad Altisent, Rishi Puri

**Affiliations:** 1 Department of Cardiovascular Medicine, Heart Vascular & Thoracic Institute, Cleveland Clinic, 9500 Euclid Avenue, J2-3, Cleveland, OH 44195 USA; 2 Hospital Clinic Barcelona, Barcelona, Spain

**Keywords:** Tricuspid Regurgitation, Tricuspid Valve Replacement, Caval Valve Implantation, Transcatheter Valve Replacement, Heterotopic Valve Replacement

## Abstract

**Purpose of Review:**

We describe the evolution of caval valve implantation (CAVI) as a treatment for severe symptomatic tricuspid regurgitation (TR) in the high surgical risk patient.

**Recent Findings:**

Surgical treatment of severe TR is often limited by the high surgical risk of the patients who tend to develop severe secondary TR. Coaptation, annuloplasty, and orthotopic replacement strategies are all limited by annular and leaflet geometry, prior valve repair, and the presence of cardiac implantable device leads. CAVI appears to be a treatment strategy for severe symptomatic TR that improves functional capacity and quality of life while also reducing edema and ascites and improving cardiac output. Chronic kidney disease is a common comorbidity of patients with severe TR; zero-contrast CAVI has been described.

**Summary:**

Severe TR is undertreated, yet common in the elderly structural heart disease population. The evolution of CAVI as a viable treatment for severe TR underscores the deleterious systemic contribution of backwards flow to morbidity and mortality. There are good safety and efficacy outcomes from registry data using the TricValve platform. Randomized controlled trials for CAVI versus medical therapy for severe TR are ongoing.

## Introduction

Severe tricuspid regurgitation (TR) is a prevalent and undertreated disease [1–4]. Isolated tricuspid valve (TV) surgery carries the highest mortality for isolated valve surgery, and patients referred for interventional therapies are often high or very high surgical risk patients. Anatomic considerations can affect candidacy for tricuspid “clipping” or transcatheter tricuspid valve replacement. Caval valve implantation (CAVI) overcomes many of the anatomic limitations of TTVI, and there are now three dedicated CAVI platforms being investigated: the TricValve, Trillium, and Unica. This review describes the evolution of CAVI along with periprocedural factors to maximize procedural success and patient outcomes. The findings and lessons from first-in-man reports, single-arm interventional experiences, and CAVI registry outcomes are described here.

### The Role of CAVI

Severe TR often marks the presence of advanced left heart or pulmonary disease and portends a worse prognosis in those with untreated left sided valvular disease [5, 6]. Hepatic, renal, and gastrointestinal dysfunction are common from prolonged exposure to elevated right-sided filling pressures. As severe TR persists, progressive RV dilatation begets more TR via tricuspid annular dilatation [7]. Eventually, frank RV failure and low-output syndrome occurs [8]. In this stage, patients with severe TR are often relegated to a palliative diuretic approach that is ultimately unable to keep pace with progressive edema, anorexia, and dyspnea.

Historically, operative mortality rates for severe isolated TR were upwards of 10% [9, 10]. Improved surgical techniques, better patient selection, and the demonstrated safety and efficacy of transcatheter techniques have led to renewed interest in earlier intervention for severe TR before the onset of irreversible RV dysfunction [9, 11, 12]. In patients undergoing surgery for left-sided valve disease, the 2020 ACC/AHA valve guidelines provide a class 2a recommendation for TV repair for non-severe TR but significant tricuspid annular dilatation or signs and symptoms of right heart failure [13]. This is based on data suggesting that concomitant tricuspid valve repair in patients undergoing mitral valve repair limits the progression of long-term TR in appropriately selected patients [14, 15]. Unfortunately, the majority of cases of severe TR come to the attention of patients and physicians late into the natural history of the disease, and patients are often no longer low-risk re-operative candidates once interventional therapy is required.

Transcatheter tricuspid valve interventions (TTVI) offer promise in the treatment of severe TR for patients that are high surgical risk or inoperable [12, 16]. The surge of interest in TTVI in the last decade follows the success of transcatheter therapies for left-sided valve disease, with early TTVI comprising the adoption of mitral transcatheter edge-to-edge repair (TEER) into the tricuspid space [17]. The U.S. Food and Drug Administration’s (FDA) recent approval of tricuspid TEER (T-TEER) followed randomized trial data demonstrating safety and efficacy of a dedicated T-TEER platform (TriClip™) in patients with severe TR [18]. Concomitant U.S. FDA approval of an orthotopic transcatheter tricuspid valve replacement (TTVR) system followed recent data indicating 30-day safety and superior 12-month clinical efficacy data for the EVOQUE™ TTVR platform compared to medical therapy alone for patients with symptomatic severe TR [19]. While these data are promising, the feasibility and procedural success of T-TEER and orthotopic TTVR depend heavily on TV anatomy and peri-procedural imaging. Excessive annular dilatation, extreme leaflet separation/coaptation gaps, RV size, and the presence of cardiac device leads can render patients suboptimal for T-TEER or orthotopic TTVR [20]. For these patients, heterotopic TTVR may prove helpful.

Table 1 provides a summary of anatomic and physiologic considerations when determining a TTVI approach. Importantly, future additional options for tricuspid valve intervention are preserved for patients that undergo CAVI up front, which is not the case in those who undergo T-TEER or orthotopic TTVR. When accounting for the anatomic and imaging factors in Table 1, we estimate that ~ 40–50% of severe TR patients are likely good candidates for T-TEER; ~50% are likely good candidates for orthotopic TTVR; and ~ 80% are likely good candidates for CAVI. Single-center experience with TTVI referrals suggest that the majority of referred patients were excluded from orthotopic TTVI trial randomization owing to unsuitable anatomy, patient comorbidities including the presence of multiple cardiac implantable device leads, and the presence of prior tricuspid repair or replacement. Ultimately, ~ 48% of the > 180 consecutive patients referred for severe TR in our large single-center experience went without an interventional therapy to specifically treat TR between 2019 and 2021 (Abushouk A et al. 2024, data in press).

**Table 1:** Considerations for method of transcatheter tricuspid valve intervention

TTVI approach	Favorable conditions	Challenging conditions
Coaptation (TEER)	• Small coaptation gap (< 7 mm ideally) • Antero- or posteroseptal TR jet location • RV lead but no leaflet obstruction	• Very large coaptation gap (> 10 mm) • Anteroposterior TR jet location • RV lead with leaflet tethering • Diffusely degenerated/thickened leaflets
Annuloplasty	• Favorable RCA course for anchoring • Predominant TR mechanism is atrial functional TR	• Large coaptation gap (> 7 mm) • Predominant TR mechanism is RV lead impingement/obstruction • Unfavorable RCA anatomy • Severe pulmonary hypertension
Orthotopic TTVR	• Large coaptation gap (> 7 mm) • Predominant TR mechanism is primary TR • History of prior tricuspid valve repair	• > Moderate RV dysfunction or a small RV; or in contrast, very large annular dilatation (≥ 60 mm • Severe pulmonary hypertension • Proximity to RCA, conduction system • Unfavorable angle between IVC and tricuspid annulus
Heterotopic TTVR (CAVI)	• Anatomy that prohibits coaptation approach, annuloplasty, or orthotopic TTVR • RV leads are not an issue • Severe RV dysfunction is acceptable but there should be elevated IVC *v*-waves and not severe pulmonary hypertension	• Unfavorable vena cava or hepatic vein anatomy • Contraindication for oral anticoagulation

TTVI: transcatheter tricuspid valve intervention; TEER: transcatheter edge-to-edge repair; TR: tricuspid regurgitation; RV: right ventricle; RCA: right coronary artery; TTVR: transcatheter tricuspid valve replacement; IVC: inferior vena cava; CAVI: caval valve implantation

### Development and Evolution of CAVI

Heterotopic TTVR – specifically caval valve implantation (CAVI) – involves implantation of a prosthetic valve at the cavo-atrial junction(s). The physiologic goal of CAVI is to reduce the systolic regurgitant backflow of TR into the vena cava and mitigate the impact of TR on hepatic, renal, and gastrointestinal congestion. Peripheral edema and ascites are expected to rapidly improve, and functional capacity is expected to increase. The initial concept of heterotopic TTVR was tested in sheep by Lauten et al. and published in 2010 [21]. After creating acute TR by damaging the ovine tricuspid valves, bovine pericardial valves were mounted onto self-expanding nitinol stents and implanted into both the IVC and SVC. Valve implantation reduced the *v*-waves and *y*-descent of the vena cava pressure waveform and increased the cardiac output back to baseline before the acute TR was created.

A description of the first-in-human experience with CAVI for severe TR was published in 2011 with the implantation of a custom-made self-expanding IVC valve at the cavo-atrial junction [22]. The index patient was not a candidate for surgical TV repair, and medical therapy was ineffective at relieving symptoms of congestion, ascites, or resting dyspnea; the patient therefore underwent compassionate-use CAVI. In this first-in-human case, the custom-made covered stent had a maximum diameter of 43 mm and a length of 70 mm; a trileaflet porcine pericardial valve was mounted with an anticipated landing zone immediately above the hepatic vein inflow. A custom-made 27-Fr catheter was used to deliver the device from right femoral venous access. Continuous venous pressure monitoring confirmed v-wave reduction in the IVC from 29 mmHg to 19 mmHg after device deployment, and 8-week follow-up with echocardiography and CT confirmed excellent prosthetic valve function. The patient was discharged and outpatient follow-up confirmed improvement of NYHA functional class from IV to III. Unfortunately, the patient died from intracranial hemorrhage 3 months later; post-mortem examination of the CAVI valve demonstrated endothelialization of the stent struts and soft pliable leaflets without evidence of thrombus or degeneration.

The early experience of compassionate-use CAVI from 2010 to 2017 was described in a case series of 25 nonoperative hospitalized patients with persistent and debilitating heart failure symptoms despite medical therapy and severe symptomatic TR [23]. Eighteen of the 25 patients received IVC-only valves (with 16 receiving a balloon-expandable Sapien XT or Sapien 3 valve following pre-stenting of the vena cava). Procedural success was high in this series, systolic caval backflow was significantly reduced in all cases, and most patients experienced improvement in NYHA functional class prior to discharge. Despite clinical outcomes being confounded by comorbidities in these very sick patients, this early series illustrated the feasibility and potential of CAVI as a treatment option for severe TR.

Following the success of the feasibility studies in compassionate-use cases, the TRICAVAL study randomized nonoperative severe TR patients to a non-dedicated balloon-expandable CAVI device (Sapien XT valve) versus medical therapy [24]. Of 28 patients in TRICAVAL, 14 underwent IVC-only CAVI using the Sapien XT valve (sizes 23, 26, or 29 mm) following pre-stenting of the vena cava. Prosthetic valve dislocation (2 cases of stent migration and 2 cases of valve embolization) and higher mortality in the CAVI group led to early termination of this study [25]. Now, CAVI with non-dedicated caval devices has largely been abandoned due to the advent and evolution of dedicated bicaval CAVI systems.

### Dedicated CAVI Systems

#### TricValve

**Fig. 1:**
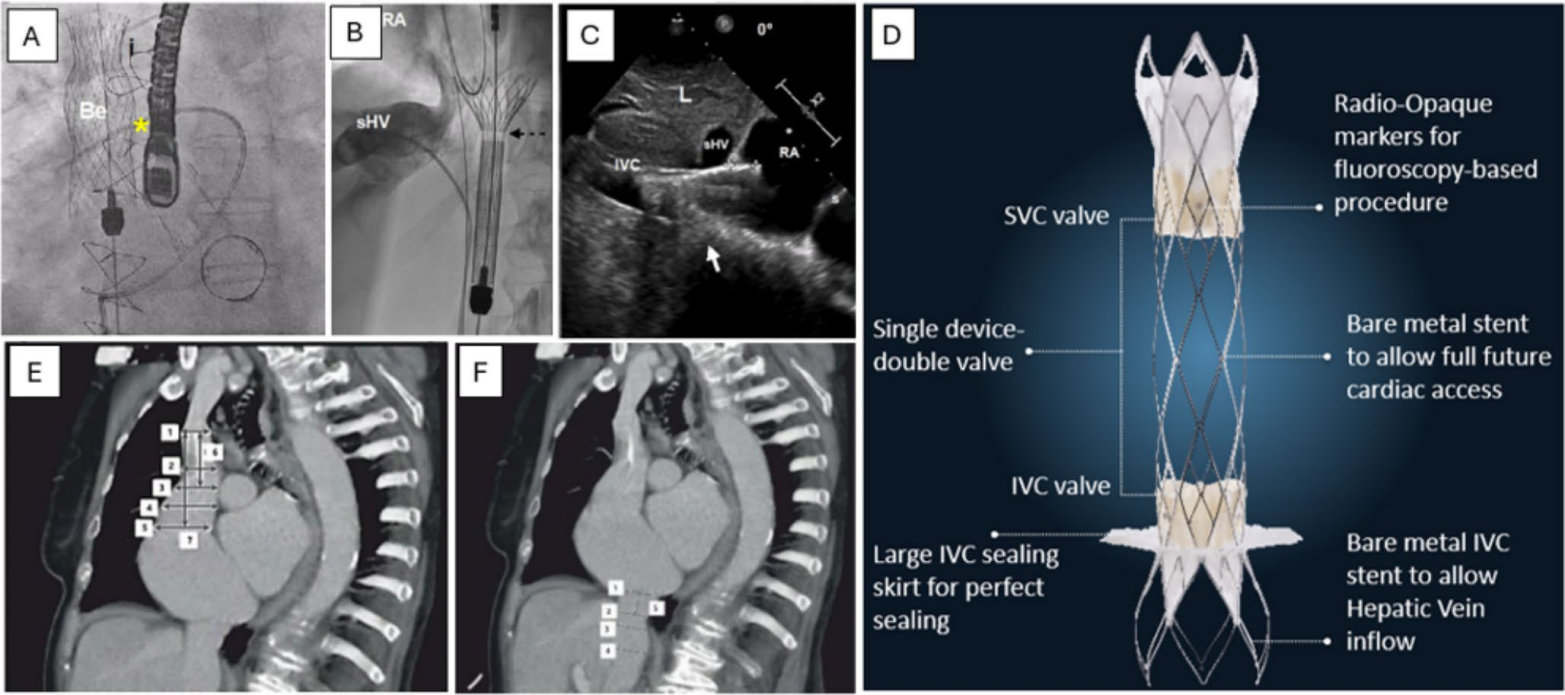
Legend. (**A**) The “belly” of the SVC stent graft should be implanted at the level of the right pulmonary artery, marked here with a Swan Ganz catheter (*star)*. (**B**) The IVC valve has a sealing skirt covering the proximal 20 mm of the stent; the radiolucent band marks where the skirt ends. (**C**) Optimal positioning should have 5 to 12 mm of the proximal edge of the IVC stent entering the RA. TEE (at 0$\:^\circ\:$ plane) shown here. *White arrow* indicates the inferior cavo-atrial junction. (**D**) Unica (Innovalve), a single-device, dual-anchoring caval stent with two bioprosthetic valves. Open struts at the right atrial face permit easier cardiac access for subsequent procedures. (Fig. 1D with permission from Innoventric.) (**E**,** F**): SVC and IVC measurements in CT sizing for TricValve. Relevant SVC diameter measurements (from top to bottom) are at the confluence with the innominate vein; at the top, middle, and bottom levels of the pulmonary artery; and at the superior cavo-atrial junction. Orthogonal lengths from innominate confluence to mid pulmonary artery level and from innominate confluence to superior cavo-atrial junction are also recorded. Relevant IVC diameter measurements (from top to bottom) are at the inferior cavo-atrial junction; the top and bottom of the hepatic vein confluence, and at 5 cm inferior to the inferior cavo-atrial junction. Orthogonal length from inferior cavo-atrial junction to the top of the hepatic vein confluence is also recorded. (**Figures A-C**,** E-F** from: J Clin Med. 2021;10:4601; https://www.mdpi.com/2077-0383/10/19/4601; Creative Commons user license https://creativecommons.org/licenses/by/4.0/) [20]. Be: belly of SVC stent graft; RA: right atrium; sHV: superior hepatic vein; IVC: inferior vena cava; SVC: superior vena cava

While feasibility data from compassionate-use case series have been described for several CAVI systems (TricValve, Tricento, and Trillium/Unica), only the TricValve platform has prospective clinical trial data published with a Conformité Européenne (CE) mark. Evolved from the first-in-human custom-made IVC valve for CAVI, the TricValve is now a bicaval platform comprising a self-expanding valve for the IVC (sizes 31, 35, 41, and 45 mm) and a self-expanding valve for the SVC (sizes 25, 29, and 33 mm). Both prosthetic valves are pre-mounted on a 27.5 Fr delivery catheter using pericardial dry-leaflet technology. Right femoral venous access is typically utilized for device delivery. With venography guidance, the belly of the prosthetic SVC valve is positioned below the innominate confluence within the SVC and immediately above the plane of the right pulmonary artery; adjustments are made for different SVC morphologies or for the presence of intracardiac device leads. In turn, the proximal edge of the prosthetic IVC valve should be positioned between the cavo-atrial junction and the confluence of the supra-hepatic vein, with overlap into the RA by no more than 10 mm to minimize paravalvular leak. Caution should be taken to not occlude the hepatic vein inflow with the 20 mm long skirt on the IVC valve prosthesis [20] (Fig. 1A and C).

The 30-day safety and 6-month efficacy data from TRICUS EURO was published in 2022 [25]. This Conformité Européenne mark trial enrolled 35 patients between 2019 and 2021 with severe symptomatic TR for TricValve implantation. Enrolled patients needed to have LVEF $\:\ge\:$40%, and estimated systolic PA pressures $\:\le\:$65 mmHg, vena caval *v*-waves $\:\ge\:$25 mmHg on right heart catheterization, and a CT scan demonstrating anatomic suitability. Those with significant renal dysfunction (sCr >3.0 mg/dL or renal replacement therapy within 4 weeks of screening) or Child-Pugh class C cirrhosis were excluded. Mean age in the enrolled cohort was 76 years and mean EuroScore II was 5.8%. Mean tricuspid annulus size was 41 mm, RV end-systolic area was 11.5 cm^2^, TAPSE was 18 mm, and RV FAC was 47.7%. Procedural success as 94% in this cohort (successful technical deployment and positioning without periprocedural major adverse events plus fall of the *v*-wave on vena cava pressure waveform after device deployment), and patients had significant improvement in quality of life by KCCQ and NYHA functional class at 6-month follow up. The most frequent early postprocedural adverse event was transient shoulder pain (28.5% of patients) suspected to be a result of phrenic nerve compression, and there was neither device-related mortality nor cardiovascular death at 6-months of follow up.

One-year follow-up for patients enrolled both in TRICUS EURO and the initial TRICUS EFS were recently published [26]. Over 95% of patients attained either higher KCCQ by $\:\ge\:$15 points, improvement in NYHA functional class to I or II, or increased 6-minute walk distance by $\:\ge\:$40 m. The heart failure rehospitalization rate was 29.5%, and 63.8% of patients had durable abolition of systolic reversal in hepatic venous flow at 1 year. There were no cases of stent fracture, conduction system disturbances, or clinically significant leaflet thrombosis among the patients studied [26]. There is emerging evidence that, despite anatomic ventricularization of the RA, CAVI can lead to significant improvements in cardiac output and potentially reverse RV remodeling in appropriately selected patients [27, 28]. Additional studies are needed to investigate these concepts further, but TricValve registry data presented by Sanchez-Relcade at EuroPCR 2024 showed reductions in mid- and basal RV diameter at 3-month follow-up after CAVI in 204 patients across 27 European hospitals. Cardiac output was preserved and IVC pressures remained low on 3-month right heart catheterization despite massive/torrential TR at baseline. There were marked reductions in peripheral edema, ascites, hospitalizations for heart failure, and diuretic dosing at 1-year follow up, with significant improvements in functional status.

Efforts are ongoing for randomized controlled trials of the TricValve platform. The multinational pivotal TRICAV study is a prospective trial to evaluate safety and efficacy of TricValve for high surgical risk patients with severe symptomatic TR with an optimal medical therapy control arm that is presently enrolling (NCT06137807). Inclusion and exclusion criteria are similar to those in the TRICUS studies. The TRICAV-1 early feasibility study is a 15-patient, 30-day follow-up single arm study currently enrolling in the U.S. across select centers, with a planned transition to TRICAV-2, a randomized controlled pivotal trial (2:1 randomization to TricValve versus medical therapy) involving > 400 patients aimed for U.S. FDA premarket approval submission.

### Trillium and Unica

The Trillium and Unica (Innoventric) devices are both self-expanding cross-caval platforms with a single device stretching from SVC to IVC. These devices offer an element of procedural simplicity with a strictly fluoroscopic approach for implantation, allowing for skin-to-skin device implant times under 10 min [29]. Both devices have designated IVC skirts to allow for complete sealing of the RA from the IVC, with the Unica system sealing against IVC diameters up to 60 mm [30]. The Trillium CAVI device is primarily a covered stent with three openings that face the RA and tricuspid valve allowing for antegrade venous return to the right heart; in contrast, the central portion of the Unica CAVI device is bare metal, permitting ease of right heart access after device implantation (Fig. 1D). The first-in-human use of the Unica CAVI system involved a 79-year-old woman who had torrential TR, right heart failure, and prior failed attempts at T-TEER. The crimped device was delivered over a 24-F capsule delivery system via the right femoral vein, with immediate reduction in IVC venous pressure and abolition of systolic venous backflow on echocardiogram after deployment. The patient experienced improvement in NYHA functional class and patient-reported KCCQ score, with no peripheral edema and no new hospitalizations at 6-month follow up [30]. CE mark studies are underway (NCT04289870).

### Clinical Considerations for CAVI

Patients being evaluated for CAVI have symptomatic severe TR and are often being referred by non-interventionalists for consideration of TTVI at large. Advantages of CAVI compared to alternative TTVI methods include its relatively shorter learning curve and shorter procedural time; specifically, the interventionalist does not need to navigate the angle from the vena cava to the tricuspid annulus when deploying caval valves. As noted in Table 1, excessive coaptation gaps or annular dilatation pose challenges for T-TEER and orthotopic TTVR, but these limitations are obviated in CAVI. Furthermore, general anesthesia and intraprocedural transesophageal echocardiography, while occasionally helpful, are not mandatory.

On the other hand, proper anatomic planning by CT is crucial for patient selection, with minimum distances from innominate vein to cavo-atrial junction and cavo-atrial junction to supra-hepatic vein being paramount to TricValve candidacy (Fig. 1E-F). The risk of device migration is also higher in those with extremely dilated IVCs or tapered and short SVCs [20]. Proper pre-procedural cross-sectional imaging with CT angiography – or MR angiography in select cases – can pay dividends in patients with comorbid chronic kidney disease who may then undergo contrast-sparing CAVI with the use of intraprocedural intravascular ultrasound [31].

As with orthotopic TTVR, patients need to be able to tolerate long-term/indefinite anticoagulation given the presence of a prosthetic tissue valve in a low-velocity system. Furthermore, because CAVI closes off the vena caval “pop-off” in severe TR, severe RV dysfunction or severe pulmonary hypertension at baseline are part of exclusion criteria for ongoing CAVI trials. Importantly, the theoretical benefit of CAVI requires the existence of a sufficiently elevated caval *v*-wave at baseline, implying an RV with sufficient systolic reserve.

When considering treatment strategies in patients with significant right heart failure and RV dysfunction, optimization of fluid status and left-sided filling pressures is crucial to device candidacy and procedural success [12]. With aggressive diuresis, changes in both hemodynamics of the RV and PA and geometry of the tricuspid annulus and vena cava can be expected; large amounts of diuresis may be required owing to the relative flatness of the Frank-Starling curve for the right ventricle [7]. These changes impact the feasibility of CAVI, CAVI device sizing, and expected post-procedural outcomes. Because CAVI closes off the vena caval “pop-off” in severe TR, RV afterload increases acutely after the procedure. In those with elevated systolic PA pressures at baseline and marginal RV function, close assessment of RV reserve with invasive hemodynamics is recommended to increase the likelihood of procedural success. In those with post-procedure RV shock, consideration should be given to RV inotropes (e.g., low-dose milrinone, levosimendan) or even right-sided mechanical support across the CAVI prosthesis in extreme circumstances [32].

## Conclusions

The landscape of TTVI is rapidly changing, and the tricuspid valve should no longer be regarded as “the forgotten valve.” That being said, TR is a powerful negative prognostic marker for all cardiopulmonary disease, and the population we currently seek to treat with novel therapies for severe TR are frequently advanced in their disease course, elderly, and burdened with a greater degree of comorbidities. The hope with CAVI is that patients who have severe TR may live with improved quality of life, experience greater freedom from rehospitalizations, and exhibit better overall function. With improved dedicated caval platforms, a quicker learning curve compared to other forms of TTVI, and prospective trials underway, the upcoming years may witness CAVI go from a method regarded as palliative to a standard treatment option alongside other modes of TTVI.

## Data Availability

No datasets were generated or analysed during the current study.

## Declarations

### Key References

Blasco-Turrión S, Briedis K, Estévez-Loureiro R, Sánchez-Recalde A, Cruz-González I, Pascual I, et al. Bicaval TricValve Implantation in Patients With Severe Symptomatic Tricuspid Regurgitation. JACC Cardiovasc Interv. 2024;17:60–72.

**This Analysis Describes the 1-year Clinical Endpoints from the TricValve single-arm Interventional CAVI Experience**.

Denby K, Spilias N, Harb SC, Kapadia SR, Puri R. Contrast-Sparing Intravascular Ultrasound–Guided Caval Valve Implantation for Severe Symptomatic Tricuspid Regurgitation. JACC Case Rep. 2023;23:102007.

**This series describes contrast-sparing techniques for CAVI that utilize IVUS, important due to the prevalence of chronic kidney disease in patients with severe tricuspid regurgitation and right heart failure.**
